# Dose to organs at risk in the upper abdomen in patients treated with extended fields by helical tomotherapy: a dosimetric and clinical preliminary study

**DOI:** 10.1186/1748-717X-8-247

**Published:** 2013-10-25

**Authors:** Sara Bresciani, Elisabetta Garibaldi, Gabriella Cattari, Angelo Maggio, Amalia Di Dia, Elena Delmastro, Domenico Gabriele, Michele Stasi, Pietro Gabriele

**Affiliations:** 1 Department of Medical Physics, Institute for Cancer Research and Treatment (IRCCS) at Candiolo, Turin, Italy; 2 Department of Radiation Oncology, Institute for Cancer Research and Treatment (IRCCS) at Candiolo, Turin, Italy; 3 Department of Neuroscence, University of Turin, Turin, Italy

**Keywords:** Upper abdominal organ at risks, Helical TomoTherapy, Extended fields, Toxicity

## Abstract

**Background:**

The aim of this work was to determine the technical feasibility and safety of extended-field radiotherapy (EF), performed by Helical TomoTherapy, in patients with positive pelvic and/or para-aortic nodes. Dosimetric data were collected and acute and sub-acute toxicities of the upper abdominal organs at risk (OAR) were evaluated.

**Methods:**

Twenty-nine patients suitable for EF irradiation for local disease and/or nodal disease in the pelvic or para-aortic area were treated. The prescription dose was 50.4/54 Gy (1.7-1.8 Gy/fraction) for prophylactic lymph nodes (N-) and 60–70.5 Gy (2–2.35 Gy/fraction) for clinically evident gross disease (N+). Modulation factor (MF), pitch and field width (FW) were chosen to optimize dose distribution and treatment duration. Dose values of PTVs and OAR were analysed. The length of the treatment field, the N + and N- volumes, and treatment duration were reported. To evaluate the safety of treatment, haematological, hepatic, renal and pancreatic functions were assessed before, during and after treatment. The median follow-up time was 17.6 months (range: 6–22 months).

**Results:**

The treatment was well tolerated and all patients but one completed treatment without interruption. Four of the 29 patients experienced G3 haematological acute toxicity (13.8%), but no patient experienced sub-acute grade G3 toxicity. Ten patients experienced G1 and three G2 acute gastrointestinal toxicity (nausea). No sub-acute gastrointestinal or renal toxicity was observed. Only one (3.7%) patient had a persistent slight increase of pancreatic enzymes and two (7.4%) patients a slight increase of hepatic enzymes six months after radiotherapy (G1 toxicity).

**Conclusions:**

With our treatment design and dose regimen, we found that EF treatment by TomoTherapy could be safely and effectively delivered with minimal acute and sub-acute toxicities in the upper abdomen area.

## Background

Extended volume irradiation for pelvis or lumbar-aortic volumes, which may be required in some stages of prostate or uterine cancer, presents difficulties when performed with conventional conformal techniques [1].

Extended field (EF) irradiation was described in the literature as early as the 1970s [2,3], but the use of conventional radiotherapy techniques has been reported to result in incomplete coverage of the target volume [4]. Furthermore, standard techniques are also associated with normal tissue complications. Indeed, with these techniques, excessive amounts of organ at risk, such as small bowel, have been included in the treatment fields, resulting in increased gastrointestinal toxicity. In addition, a large volume of bone marrow may be irradiated, leading to hematologic count depression and untoward toxicity and treatment interruption, especially in patients requiring concurrent chemotherapy [5]. Furthermore, dose escalation to grossly involved nodes has been limited by concerns of toxicity to abdominal critical structures, particularly in upper abdomen, such as liver or kidneys [6].

Several reports have described the advantages of intensity modulated radiation therapy (IMRT) over 3D-CRT for EF treatments; the dosimetric advantage of IMRT may improve the therapeutic outcome and reduce early and late toxicity [7-9]. Compared with 3D-CRT and IMRT by linac, Helical TomoTherapy (HT) allows EF to be delivered without field junctions. HT also delivers a more highly conformal dose distribution due to a greater number of independent beam directions and facilitates irradiation of large fields, with benefit especially for patients requiring para-aortic lymph node irradiation [10]. However, the potential clinical benefit of HT over IMRT is still being investigated [11,12]. Furthermore, the body of literature relating radiation doses to risk of abdominal organs toxicity (stomach, small bowel, liver, kidneys, pancreas and spleen) is small compared with the amount of data published on RT effects in some others organs, such as rectum [13]. Indeed, although previous studies have demonstrated that EF IMRT is safe and effective with a low incidence of toxicities [7], very few studies have reported relations of the dose in the upper abdominal organs to normal tissue toxicity, especially in protocols involving dose escalation to involved nodes.

The aim of this study was to perform a dosimetric and toxicity analysis of HT plans in EF RT for pelvic and para-aortic nodal regions with dose escalation to positive nodes. We are reporting here our initial experience for patients with locally advanced/recurrent prostate, endometrial and cervical cancer by HT, focusing on the technical feasibility of EF irradiation through the analysis of dose-volume histogram (DVH) parameters and their correlation to toxicities, including assessments of organ function by complete blood counts and the laboratory tests.

## Methods

Between October 2010 and December 2012, 29 patients were treated with extended-field IMRT (EF-IMRT) by HT units. The series included 17 patients with nodal recurrences of prostate cancer, 4 patients with very high-risk prostate cancer, 5 patients with stage II-III of cervical cancer and 3 patients with postoperative stage II-III endometrial carcinoma. The intent of treatment was radical in patients with prostate cancers, both primary and recurrent, and in cervical cancer patients, and adjuvant in endometrial cancers. Median age was 65.8 years (range 40–84 years).

All patients with prostate cancer (both primary and recurrent) received concomitant hormonal therapy. Patients affected by cervical cancer received concomitant chemotherapy with weekly cisplatinum. The three patients with operated endometrial cancer received adjuvant chemotherapy with carboplatinum and taxol.

Before radiotherapy each patient underwent a CT-simulation scan (BigBore Aquilion 16@Toshiba) with 3-mm slice thickness from the supra-diaphragmatic level to 5 cm below the ischial tuberosities. Localization marks were placed on anterior and lateral sides of the patients between the fourth and the fifth lumbar vertebra. Patients were positioned supine with arms up, immobilization systems used to fix extremities and pelvis were Harm Shuttle and Pro-Step (Q-Fix, Avondale, PA, USA), respectively. Two patients with recurrent prostatic tumour underwent 18F-choline PET/CT virtual simulation. All patients with cervical and endometrial cancer received an 18F-FDG PET/CT virtual simulation. PET images were retrieved on a Pinnacle3 treatment planning system (Philips Healthcare, Madison, Wisconsin, USA) and matched with the CT images for the tumour volume contouring.

Target volumes and normal tissues were contoured on Pinnacle workstation, according to the International Commission on Radiation Units and Measurements (ICRU) Report No. 62 and 83 recommendations [14,15]. The gross tumor volume (GTV), the clinical target volume (CTV) and lymph node regions (common, internal and external iliac, and para-aortic regions) were contoured. The planning target volume (PTV) was created by expanding uniformly the CTV volumes in order to account for organ motion and setup uncertainty: for prostate gland and seminal vesicles, an anisotropic margin of 0.8 cm was added, except for posteriorly where the margin was 0.6 cm. For pelvic and para-aortic prophylactic nodes we added an isotropic margin of 0.5 cm in all directions and for involved nodes we added a tailored margin of up to 0.7 cm, depending on proximity of small bowel or other critical structures.

The rectum (defined from the sigmoid flexure to the anus), sigma-colon, bladder and femoral heads, anus, penile bulb, also small bowel (defined as intestinal cavity), kidneys, pancreas, stomach, spleen, ureter, and the liver were contoured on all patients for dosimetric and toxicity evaluation. The small bowel portion inside PTV N- and PTV N + was designated as “small bowel in” and was used during optimization to achieve sufficient dose sparing of the OAR.

**Figure 1:**
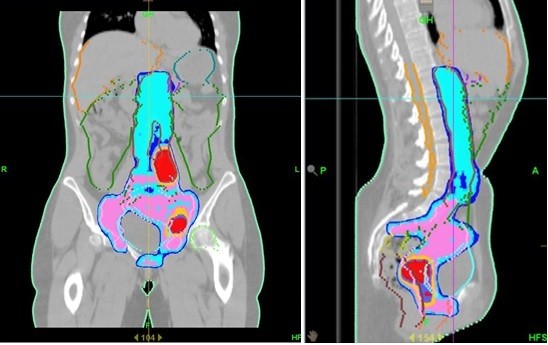
**Example of extended field irradiation for cervical carcinoma.** Isodose distribution for sagittal and coronal projections through the patient’s midline is provided (red = 66Gy, pink = 54Gy, blue = 51Gy).

The dose prescription for the EF-IMRT plans was 51–54 Gy (1.7-1.8 Gy/fraction) for prophylactic lymph nodes and 60/66 Gy (2–2.2 Gy/fraction) to PTV N + in the para-aortic or pelvis chain, with a simultaneous integrated boost (SMART technique irradiation). Lymph nodes dose prescription depended on their localization: for PTV N + lymph nodes that overlap the small bowel loops, the dose prescription was 60 Gy (Figure 1).

CT data-sets and structures were transferred from Pinnacle3 to the Tomotherapy Planning Workstation. Parameters specified as part of the optimization/dose calculation process are pitch, field width (FW) and modulation factor (MF). By increasing the FW and reducing the pitch and MF we can significantly reduce the treatment time. For optimum dose conformality, small FW and pitch and high MF should be employed, at the expense of longer treatment times. In clinical practice, the selection of FW, pitch and MF represents a compromise between excessively long treatment times (with highly conformal delivery) and shorter treatment times (with some loss of conformality ). Prior to optimization, dose volume constraints, overlap, importance and penalty factors for target and critical structures were assigned. Accuracy of setup was verified daily by megavoltage CT imaging (MVCT).

Patients were evaluated weekly during the course of radiotherapy to assess acute toxicity. Acute toxicity was defined as those occurring within 90 days of competition of radiation therapy. Hematological, hepatic, renal and pancreatic functions were evaluated by complete blood count and laboratory tests before, during and after treatment. Toxicities were graded according to the National Institute Common Toxicity Criteria for Adverse Events (CTCAE), version 3.0 scale.

Dose-volume histograms (DVHs) of the PTVs and the critical normal structures were analysed. For PTVs, we evaluated the average dose and the dose delivered to 95% of Volume (D_95%_).

The length of the treatment field, the volume (cc) of the positive lymph nodes (N+) and prophylactic lymph nodes (N-) were also evaluated. For OAR, the mean and maximum dose of small bowel, pancreas, spleen, stomach, kidneys and liver were examined. V_45_ of small bowel was registered according to QUANTEC recommendations [12]. Our dose-volume constraints are summarized in Table 1. Finally, when we adopt a new technology careful considerations are necessary, particularly in a field where the incidence of late second cancers is becoming a dominant concern. For this reason and for a better assessment of the safety of the EF irradiation by TomoTherapy we evaluated also the low dose distribution deposited in the critical structures, in terms of V_5Gy_, V_10Gy_, V_15Gy_ of the body [16].

**Table 1:** Normal tissue tolerance

**Critical structure**	**Volume**	**Dose/volume**	**Toxicity rate**	**Toxicity endpoint**
Liver	Mean	<30-32 Gy	<5%	RILD (in normal liver function)
Kidney, bilateral	Mean	<15-18 Gy	<5%	Clinical dysfunction
Kidney, bilateral	Mean	<28 Gy	<50%	Clinical dysfunction
Kidney, bilateral	V12	<55%	<5%	Clinical dysfunction
Kidney, bilateral	V20	<32%	<5%	Clinical dysfunction
Kidney, bilateral	V23	<30%	<5%	Clinical dysfunction
Kidney, bilateral	V28	<20%	<5%	Clinical dysfunction
Stomach	D100	<45 Gy	<7%	Ulceration
Small bowel (peritoneal cavity)	V45	<195 cc	<10%	Grade 3+ toxicity

The correlation between gastrointestinal, liver and pancreatic toxicities and dosimetric parameters were investigated in univariate analysis (logistic regression).

## Results

In most cases, plan parameter values used for these treatments were FW = 2.5 cm, pitch = 0.287 cm and MF = 2.2. In two cases we used a FW of 5 cm to reduce treatment time. The value of these parameters were chosen in order to produce a treatment that could be delivered in a reasonable length of time (*<*15 min). In one re-treatment case we used a FW of 1.05 cm because the new target was just superior to a previously treated PTV and the smaller field has a smaller penumbra in longitudinal direction. The median actual MF was 1.8 (range: 1.3-2.6). The average length of treatment was 32.5 cm. Dose calculation grid was always set to fine mode. Median treatment time was 660 sec (range: 306–912 sec). The overall pretreatment daily setup procedure with control of positioning accuracy was performed in 890 sec (range: 530–1050 sec).

Each planning target volume was covered by 95% of the prescribed dose and a steep dose gradient was obtained in the direction of the abdominal OARs. Excellent PTV coverage was obtained: in general, the mean value of D95% for PTVs of primary targets was 96.5%, ranging between 94% and 98%. Mean absolute dose and D_95%_ were 65.3 ± 3.5 Gy and 63.7 ± 3.4 Gy to PTV_N+_, 54.5 ± 2.1 Gy and 52.2 ± 2.0 Gy to PTV_N-,_ respectively. We irradiated 1–6 positive pelvic and/or lumbar-aortic nodes simultaneously, with a mean volume of 76.6 ± 48.3 cc. Mean volumes of irradiated prophylactic nodes was 770.6 ± 307.1 cc.

Mean doses to organs at risk are shown in Table 2.

**Table 2:** Mean doses and standard deviations to organs at risk

**Organs at risk**	**D**_**mean **_**(Gy)**	**D**_**max **_**(Gy)**
Pancreas	28.1 ± 6.4	49.9 ± 9.2
Spleen	10.3 ± 6.5	25.8 ± 15.7
Stomach	16.1 ± 5.6	46.3 ± 10.2
Liver	10.0 ± 9.6	49.2 ± 11.8
Right kidney	11.8 ± 3.1	27.4 ± 7.3
Left kidney	13.5 ± 7.5	35.6 ± 12.9

**Figure 2:**
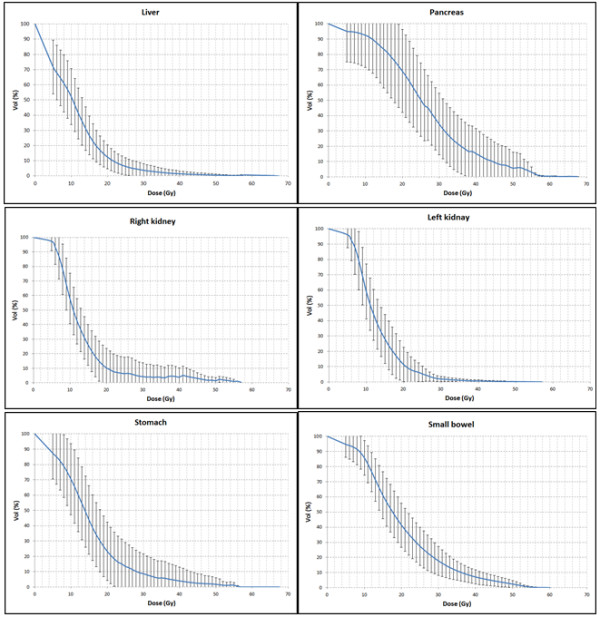
**Average DVHs of the patients.** The average DVHs of the pancreas, stomach, liver, kidneys and small bowel (intestinal cavity).

The mean volume of small bowel that received more than 45 Gy (V_45_) was 157 ± 83 cc (4.3 ± 3.7%). Maximum dose to small bowel was less than 60 Gy and the mean V_55_ was 1.8 ± 2.2cc (0.4 ± 0.5%). No patients failed to meet QUANTEC published dose objectives for the rectum, bladder, femoral heads, liver, kidney and spinal cord [17]. Only two patients treated for postoperative endometrial cancer exceeded the QUANTEC constraint for the intestinal cavity with a V_45_ > 230 cc instead of < 195 cc. The mean DVH curves for liver, pancreas, left and right kidney, stomach and small bowel are plotted in Figure 2.

The mean volume ± standard deviation of body that received more 5, 10 and 15 Gy were 22587 ± 4856 cc, 18109 ± 4344 cc and 12287 ± 3081, respectively.

### Acute and sub-acute toxicity

The median follow-up time was 17.6 months (range: 6–22 months). Overall the treatment was well tolerated: all patients but one completed treatment without interruption. One patient who received concurrent chemotherapy for cervical carcinoma required a break of 6 days due to severe urinary infection. Of these 29 patients, 10 (34.5%) experienced G1 and 3 (10.3%) G2 acute gastrointestinal toxicity (nausea). Acute hematological toxicity was the following: G1 in 7 patients (24.1%), G2 in 4 patients (13.8%; 2 of these patients received chemotherapy, one concurrently, and 2 received only hormonal therapy), G3 in 4 patients (13.8%; all of whom received chemotherapy, 3 concurrently). In 3 (10.3%) patients we observed an early slight increase of pancreatic enzyme (G1 acute toxicity) and in 5 (17.2%) patients an early slight increase of hepatic enzymes (G1 acute toxicity). No acute renal toxicity was observed. Table 3 summarizes mean doses and standard deviations to organs at risk for patients with toxicity. Table 4 summarizes acute toxicity as a function of the patient, disease, and treatment characteristics (i.e. history of prior RT or surgery).

**Table 3:** Mean doses and standard deviations to organs at risk for patients with toxicity

**Organs at risk**		**G1 acute/subacute**	**G2 acute/subacute**	**G3 acute/subacute**
Pancreas	D_mean_ (Gy)	32.0 ± 8.2/37.8	/	
	D_max_ (Gy)	61.7 ± 8.6/67.1		/
Liver	D_mean_ (Gy)	10.8 ± 3.1/11.6 ± 2.2	/	
	D_max_(Gy)	45.8 ± 12.5/54.4 ± 2.1		/
Small bowel	V_45_(cc)	168 ± 59	130 ± 84	158

**Table 4:** Acute toxicity as a function of patient and disease characteristics

**Patient characteristics**		**Acute GI toxicity**		**Acute hematological toxicity**		**Acute epatobyliary toxicity**		**Acute pancreatic toxicity**		**Acute renal toxicity**	
	total	G0-G1	≥G2	G0-G1	≥G2	G0-G1	≥G2	G0-G1	≥G2	G0-G1	≥G2
Total	29	26	3	21	8	29	0	29	0	29	0
Median age (y)	65.1	66.1	54	67.9	57.6	65.1	0	65.1	0	65.1	0
Primary prostate cancer	4	3	1	3	1	4	0	4	0	4	0
Nodal recurrence of prostate cancer	17	17	0	16	1	17	0	17	0	17	0
Postoperative endometrial cancer	3	3	0	1	2	3	0	3	0	3	0
Primary cervical cancer	5	3	2	1	4	5	0	5	0	5	0
History of abdominal surgery	15	15	0	12	3	15	0	15	0	15	0
History of prior RT	13	13	0	12	1	13	0	13	0	13	0
Paraaortic RT	10	10	0	9	1	10	0	10	0	10	0
Paraaortic + pelvic nodes RT	19	16	3	12	7	19	0	19	0	19	0
Intracavitary brachytherapy	5	5	0	1	4	5	0	5	0	5	0
Concurrent chemotherapy	7	6	1	2	5	7	0	7	0	7	0

About sub-acute toxicity, no gastrointestinal or renal toxicity was observed. G1 hematological toxicity occurred in 1 (3.5%) patient and G2 in 2 (6.9%) patients. Only one (3.5%) patient had a persistent slight increase of pancreatic enzyme and 2 (6.9%) patients a slight increase of hepatic enzymes six months after radiotherapy (G1 toxicity). All patients with hematological, pancreatic and hepatic toxicity received chemotherapy during radiation treatment. Acute and sub-acute toxicities are summarized in Table 5.

**Table 5:** Acute and sub-acute toxicity

**Toxicity type**	**Grade 0**	**Grade 1**	**Grade 2**	**Grade 3**	**Grade 4**	**Grade 5**
*Acute*						
Gastrointestinal	16	10	3	0	0	0
Hematological	14	7	4	4	0	0
Epatobyliary	24	5	0	0	0	0
Pancreatic	26	3	0	0	0	0
Renal	29	0	0	0	0	0
*Sub-acute*						
Gastrointestinal	29	0	0	0	0	0
Hematological	26	1	2	0	0	0
Epatobyliary	27	2	0	0	0	0
Pancreatic	28	1	0	0	0	0
Renal	29	0	0	0	0	0

Performing univariate analysis, we did not find any correlations between V_45_ of small bowel and gastrointestinal toxicity (p = 0.28), between liver toxicity (increase of amylases value) and D_mean_ (p = 0.55) and D_max_ (p = 0.54).

Univariate analysis showed a statistically significant association (p = 0.029) between the development low sub-acute pancreatic toxicity and D_max_, but no statistically significant association with D_mean_ (p = 0.11). In the case of acute toxicity, we did not find correlation for both D_max_ (p = 0.06) and D_mean_ (p = 0.34).

**Figure 3:**
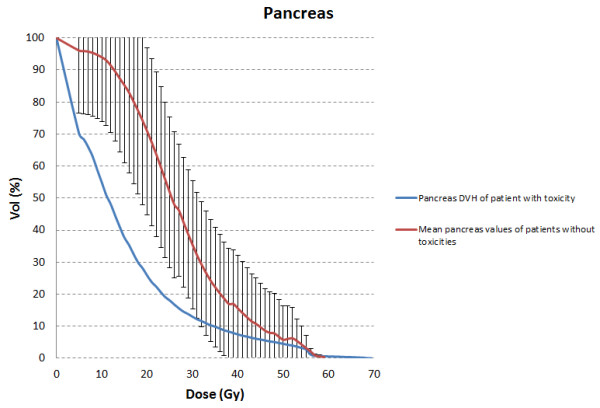
**DVH of the pancreas.** The DVH of the pancreas of patient that experienced G1 pancreatic toxicities.

In more details, about pancreatic toxicity, the only one patient that experienced G1 acute and late pancreatic toxicity showed a pancreatic dose greater than 60 Gy (D_1cc_ = 67.8 Gy). The DVH of this patient is reported in Figure 3. The values of DVH for pancreas were evaluated by comparing the mean relative volumes at selected doses values between the patient with toxicity and the others 28 without toxicities. Independent samples 2-sided t-tests were performed at each 10 Gy dose level; no p values reached statistical significance (p > 0.05), until 60 Gy. The only patient whose pancreas dose exceeded 60 Gy was the patient with toxicity.

With a mean follow up of 17.6 months the outcome of the treated patients is the following: all patients but one (partial response) had a complete response in the irradiated regions; at the moment 16 patients (55.2%) are living without disease (NED); 12 patients (41.4%) are living with disease (LWD) and 1 patient (3.5%) is died, for disease. No patients had loco-regional recurrences.

## Discussion

In the present study we analyzed the feasibility and dosimetry for upper abdominal organs at risk of EF-IMRT treatments with HT. The safety of treatment was evaluated in terms of acute and sub-acute toxicities. Dose volume constraints, laboratory tests and complete blood count were the main focus of the evaluation.

Historically, anteroposterior-posteroanterior (AP-PA) or 4-field techniques were used for EF, but the treatment was not without significant toxicity (e.g., RTOG 7920 demonstrated grade 4 to 5 GI toxicity rates of 8% with AP-PA EF-RT [17]). With standard RT techniques, dose escalation to an extended field was limited by the normal tissue tolerances.

To reduce exposure of abdominal organs at risk, in the past few years IMRT was adopted to irradiate extended fields. Several studies have assessed to treatment of the whole pelvis using IMRT (IM-WPRT), but relatively few have addressed extended-field IMRT (EF-IMRT). Kidd *et al*. demonstrated a 5.2% rate of grade ≥3 GI toxicity among cervical cancer patients receiving IMRT, of whom 13% had EF-IMRT, significantly lower than the 10.7% rate associated with conventional RT [18].

The general advantage of EF-IMRT irradiation on PTV coverage and homogeneity has been well documented, but few papers describe dose escalation in EF performed by TomoTherapy [11]. To the best of our knowledge, the advantage of IMRT on normal tissue sparing, in particular the organs of the upper abdomen, is only hypothesized and not quantified. For exemple, Portelance et al. [7] described the reduction of small bowel, rectum and kidney dose with Linac-based IMRT *vs* conventional treatment. At a dose of 45 Gy, the percentage of small bowel receiving the prescribed dose (45 Gy) with the IMRT technique varied as a function of technique as follows: four fields (11.01 ± 5.67%); seven fields (15.05 ± 6.76%); nine fields (13.56 ± 5.30%). These percentages were all significantly lower than those obtained with 2 AP-PA fields (35.28 ± 13.84%) and 4 box fields (34.24 ± 17.82%) (p < 0.05). Even though our prescription dose was higher than 45 Gy, our dosimetric result with TomoTherapy for the amount of small bowel receiving 45 Gy was 4.3 ± 3.7%, lower than IMRT and on average 87% lower than the AP-PA technique. As regards dose homogeneity and target coverage, we obtained good results with the EF-IMRT technique, without hot and cold spot regions typical of the 3D treatments (no patient with D_max_ > 107%).

The results of our analysis are particularly striking when considering the pancreas, liver and kidney, even if is not possible to perform a comparison with literature data because of the absence, to our knowledge, of a similar analysis for these organs at risk. In one report, using at least 45 Gy to the para-aortic nodes, no patients experienced acute grade ≥3 GI toxicity [7]. In a second study, EF-IMRT with dose escalation to 55 to 60 Gy administered to involved nodes along with concurrent cisplatin resulted in G3 acute and late GI toxicity rates of 2.8% and 5.6%, respectively.

As a whole, when considering the target coverage improvement, the OAR sparing capability and the ease of execution and delivery time, the use of the EF-IMRT technique shows a definite improvement in performance and safety. Our dose escalation schedule did not increase acute toxicity. We believe that clinical implementation of EF-IMRT with Tomotherapy decreases the normal tissue complications, especially when chemotherapy is added and pelvis/para-aortic lymph nodes are grossly involved, and a dose escalation protocol is required. On the other hand, a controversial aspect to be considered, especially in young patients, is the risk of induction of secondary malignancies which may result from larger low dose tissue volumes with TomoTherapy *vs* Linac-based [16].

In summary, our data show that, in the treatment of large fields, normal tissue sparing with IMRT by TomoTherapy allows very low acute and sub-acute toxicity. To date no G3 sub-acute toxicity has been observed. Moreover we confirm prior reports by showing that severe acute toxicity (G3) was observed only in patients that received concomitant chemotherapy. Given the simplicity of the EF-IMRT technique performed with TomoTherapy and the large PTVs considered, the obtained results are encouraging.

## Conclusions

With our treatment design and dose regimen, even with dose escalation up to 66 Gy (2.2 Gy/fraction) to involved nodes in para-aortic and pelvic nodal regions, we found that EF IMRT by TomoTherapy could be delivered with minimal acute and sub-acute toxicities in the upper abdomen area. Dosimetric analysis among our patient set did not establish any specific dose-volume relationships for GI and liver toxicities, but showed that high dose (>60 Gy) could be correlated with pancreatic toxicity.

Our results indicated that EF-IMRT by HT seems to be a safe treatment approach: therapy was well tolerated and there were no sub-acute Grade 3 and 4 toxicities. No patient died due to treatment toxicity. In our study, no patient to date has exhibited any renal toxicity and no patient has reported hepatic and/or pancreatic toxicities greater than G1. Longer follow-up is required to validate these favorable long-term toxicity findings.

## Competing interests

All authors declare no conflict of interests.

## Authors’ contributions

All authors were responsible for patients treatments and care. SB, EG and GC wrote the manuscript. DG collected the patients’ data. All authors helped, read and approved the final manuscript.
